# The efficacy of different biomarkers and endpoints to refine referrals for suspected prostate cancer: the *TARGET* study (*T*iered integr*A*ted tests for ea*R*ly dia*G*nosis of clinically significant Prostat*E T*umours)

**DOI:** 10.1186/s12916-024-03667-7

**Published:** 2024-10-08

**Authors:** Artitaya Lophatananon, Kenneth R. Muir, Vincent J. Gnanapragasam

**Affiliations:** 1 Division of Population Health, Health Services Research & Primary Care, University of Manchester, Manchester, UK; 2 Division of Urology, Department of Surgery, University of Cambridge, Cambridge, UK; 3 Cambridge Urology Translational Research and Clinical Trials Office, Addenbrooke’s Hospital, Cambridge Biomedical Campus, Cambridge, UK

**Keywords:** Prostate cancer, Early detection, PSA, PSA density, Free Total PSA, Polygenic risk scores, Prostate Health Index (*phi*), MRI, Biopsy, Cambridge Prognostic Groups

## Abstract

**Background:**

The majority of men referred with a raised PSA for suspected prostate cancer will receive unnecessary tertiary investigations including MRI and biopsy. Here, we compared different types of biomarkers to refine tertiary referrals and when different definitions of clinically significant cancer were used.

**Methods:**

Data and samples from 798 men referred for a raised PSA (≥ 3 ng/mL) and investigated through an MRI-guided biopsy pathway were accessed for this study. Bloods were acquired pre-biopsy for liquid biomarkers and germline DNA. Variables explored included PSA + Age (base model), free/total PSA (FTPSA), Prostate Health Index (*phi*), PSA density (PSAd), polygenic risk score (PRS) and MRI (≥ LIKERT 3). Different diagnostic endpoints for significant cancer (≥ grade group 2 [GG2], ≥ GG3, ≥ Cambridge Prognostic Group 2 [CPG2], ≥ CPG3) were tested. The added value of each biomarker to the base model was evaluated using logistic regression models, AUC and decision curve analysis (DCA) plots.

**Results:**

The median age and PSA was 65 years and 7.13 ng/mL respectively. Depending on definition of clinical significance, ≥ grade group 2 (GG2) was detected in 57.0% (455/798), ≥ GG3 in 27.5% (220/798), ≥ CPG2 in 61.6% (492/798) and ≥ CPG3 in 42.6% (340/798). In the pre-MRI context, the PSA + Age (base model) AUC for prediction of ≥ GG2, ≥ GG3, ≥ CPG2 and ≥ CPG3 was 0.66, 0.68, 0.70 and 0.75 respectively. Adding *phi* and PSAd to base model improved performance across all diagnostic endpoints but was notably better when the composite CPG prognostic score was used: AUC 0.82, 0.82, 0.83, 0.82 and AUC 0.74, 0.73, 0.79, 0.79 respectively. In contrast, neither FTPSA or PRS scores improved performance especially in detection of ≥ GG3 and ≥ CPG3 disease. Combining biomarkers did not alter results. Models using *phi* and PSAd post-MRI also improved performances but again benefit varied with diagnostic endpoint. In DCA analysis, models which incorporated PSAd and *phi* in particular were effective at reducing use of MRI and/or biopsies especially for ≥ CPG3 disease.

**Conclusion:**

Incorporating *phi* or PSAd can refine and tier who is referred for tertiary imaging and/or biopsy after a raised PSA test. Incremental value however varied depending on the definition of clinical significance and was particularly useful when composite prognostic endpoints are used.

**Supplementary Information:**

The online version contains supplementary material available at 10.1186/s12916-024-03667-7.

## Background

Prostate cancer has a high incidence but a relatively low mortality rate [1]. To date, screening studies have failed to show a clear benefit of survival against the counter-balances of over-diagnosis and over-treatment [2]. While this paradigm has been improved with the introduction of MRI (magnetic resonance imaging) pre-biopsy, it remains the case that many men need to be tested and referred to a tertiary centre before scans and biopsies are done to confirm or refute if a significant cancer is present [3]. Indeed, it is estimated that for every man diagnosed with a significant cancer, up to 2 others will have unnecessary referral and investigations [3, 4]. Natural history studies have shown that almost all men will develop cancer foci in the prostate with age but relatively few go onto cause morbidity [5]. Our understanding of what is a prognostically important cancer has also evolved greatly and the concept of not detecting cancers too early is now widely accepted [6].

Current approaches to early detection have tended to view men as a group from which the one at risk needs to be picked out and referred. The key conundrum is the balance between detecting cancer too early for which treatment is not (or will ever be) needed and hence confer no survival gain but, on the other hand, not detecting it too late when curative treatment is not possible, all within the context of a naturally growing prostate gland and ageing male. Whatever the approach, cost and cost–benefit and linking with survival outcomes are going to be key for any prostate cancer early detection programme in an ever more expensive health care environment [7]. These aspects argue strongly for a more rational and tiered strategy to the use of investigations and referrals.

Hitherto, groups working in early detection have generally fallen into separate camps, e.g. using SNP baseline population testing, bio-marker panels or imaging tests to visualise lesions [8–10]. None have gained traction to replace the simple PSA test. Thus, PSA will likely remain the first and most important baseline test, and this is the case in many current renewed efforts to re-evaluate screening. Here, we tested the incremental value of different types of biomarkers in addition to PSA to detect significant cancer and how performance may vary depending on the definition of ‘clinical significance’. Our goal was to derive the optimal sequence or testing tiers to (i) reduce the overall investigated population in men with a raised PSA, (ii) refine or reduce use of imaging and/or biopsies and (iii) explore the impact of using different definitions of clinically significant disease.

## Methods

### Patients

Data and samples from men referred for a raised PSA (≥ 3 ng/mL), investigated through an MRI-guided biopsy pathway, and who consented to participate in the DIAMOND biomarker study (research ethics 03/18) were accessed for this study (period from 2013 to 2019). All men had pre-biopsy MRI, Likert scores assigned and image-guided and systematic biopsies (or systematic biopsies only if no target seen) with resultant accurate disease characterisation. All men were biopsy naïve. Diagnostic outcomes of any cancer and significant cancers were recorded as well as a benign outcome. Different diagnostic endpoints for significant cancer (≥ grade group 2 [GG2], ≥ GG3, and composite prognosis group ≥ Cambridge Prognostic Group 2 [CPG2] and ≥ CPG3) based on the UK NICE guidelines were tested [11]. The Cambridge Prognostic Groups are a composite prognostic stratification system that has been shown to outperform older definitions of low, intermediate and high-risk and more accuracy predict prognosis from a diagnosis of prostate cancer [6, 11].

### Biomarkers studied

Research bloods were acquired pre-biopsy for liquid biomarkers and germline DNA. Variables explored included polygenic risk score (PRS), PSA (ng/mL) + age (base model), free/total PSA (FTPSA), Prostate Health Index (*phi*), PSA density (PSAd) and MRI (≥ LIKERT 3). The added value of each biomarker to the base model was evaluated using regression models and AUC.

#### Polygenic risk scores

Genotyping was carried out using Illumina Onco-array of ~ 500 k SNPs (Single-Nucleotide Polymorphism). Data quality control was carried out by filtering out any SNPs with mean allele frequencies (> 0.001) and Hardy–Weinberg equilibrium (*p* > 1E − 10). Samples were also checked for missingness (≤ 1%) and sex (male sex with no chromosome aneuploidy and no discrepancy between self-reported and genetic sex). PRS was derived from a panel of 269 SNPs previously published [12]. For missing SNPs, we carried out imputation from Chr1 to Chr X using the Michigan Imputation platform. Total SNPs included in our analyses were 245 SNPs. The genetic data file (PED and MAP files) was converted into STATA to generate a single weighted polygenic risk score (PRSj = ∑*Niβi* × dosageij), where *N* was the number of SNPs in the score, *βi* is the effect size (or beta) of variant *i* and dosageij is the number of copies of SNP *i* in the genotype of individual *j*. For each outcome, a standardised score was generated by dividing the raw score by the SD in control group (no cancer or benign group).

#### PSA, FTPSA and psad

DiaSorin Liaison assays were used for total and free PSA assays and performed by the NIHR Cambridge Biomedical Research Centre, Core Biochemistry Assay Laboratory. PSA density (PSAd) was calculated using MRI-defined prostate volumes using the ellipsoid formula.

#### *p*hi

*phi* assays were handled according to the manufacturer’s recommendations (Beckman Coulter). Samples were centrifuged and frozen at − 80 °C within 3 h before dispatch to a central laboratory and performed on a Beckman Coulter Access Autoanalyzer. Quality assurance samples were analysed before and after each batch to ensure the validity of the results. All quality control results were within Beckman Coulter’s target ranges.

#### MRI

MRI on 1.5-T or 3-T systems with multi-channel surface phased array coils were performed including standard anatomical and functional imaging (diffusion weighted and contrast enhanced). Image acquisition and processing was performed in accordance with local standard clinical protocols. Sequences were evaluated and scoring was performed according to Prostate Imaging Reporting and Data ver. 2 (PI-RADS version 2) and using a Likert scale (as this is the standard practice in our department). Likert 1–2 lesions were considered MRI negative for this study, and positive lesions were denoted as 3–5, the latter denoted as MRI Likert ≥ 3. The quality of our MRI programme in our unit has been previously documented [3].

### Statistical analysis

Data analysis was carried out using STATA 15 (StataCorp. 2017. Stata Statistical Software: Release 15. College Station, TX: StataCorp LLC.). For biomarkers except *Phi*, we imputed missing values and arbitrarily created 10 imputation datasets. We used *mi* command to generate imputed datasets and ran logistic regression and obtain *mi* estimates AUC. For *phi* (*n* = 284 men), logistic regression was performed using complete case analysis and subsequently derived AUC for each fitted model. To ensure no selection bias, comparison of the *phi* sub-cohort versus the rest of the cohort was done and did not show any differences in baseline attributes. Decision curve analysis to assess net reduction of imaging/biopsy was carried out using *dca* command.

## Results

### Cohort demographics

Data were available for analysis in 798 men. The median age was 65 years, and median PSA and prostate volume were 7.13 ng/mL and 43.9 mL respectively (Table 1). Overall, any cancer was detected in 76.5% (611/798) of men. Depending on the definition of clinical significance, ≥ grade group 2 (GG2) cancer was detected in 57.0% (455/798), ≥ GG3 in 27.5% (220/798), ≥ Cambridge Prognostic Group 2 (CPG2) in 61.6% (492/798) and ≥ CPG3 in 42.6% (340/798) of men. 2.5% (20 men) were already metastatic at diagnosis. The distributions of grade groups, clinical stage and CPG are shown in Table 1. The biomarkers tested in this study were evaluable for the vast majority of men expect for *phi* which was available in a subset of 284 men and FTPSA available in 678 (Table 2). Median polygenic risk score (PRS) was 20.6, PSA density (PSAd) 0.16 ng/mL^2^, free/total ratio 0.11 and *phi* score 41.9. MRI Likert score was considered an independent variable using a Likert score of ≥ 3 as a positive scan (Table 2). Only 2 men were not able to have MRI in this study. Notably, over a quarter of men had no visible lesion on MRI (28.7%) affirming that this cohort was not particularly selected or biased towards men with a positive MRI.

**Table 1:** Demographics of the study population. PSA, prostate-specific antigen. Grade group refers to ISUP grade group. Cambridge Prognostic Group, NICE-recommended prognostic classification system [11]. * Numbers and percentages are with reference to cancer-diagnosed cohort

	Cohort, *n* = 798
Age (years)	
Median	65
Interquartile range	59–69
PSA (ng/mL)	
Median	7.13
Interquartile range	5.3–20.7
Prostate volume (mL)	
Median	43.9
Interquartile range	32–62.7
Diagnosis	
Benign	187 (23.5%)
Cancer	611 (76.5%)
Grade group*	
GG1	156 (25.2%)
GG2	235 (38.4%)
GG3	99 (16.2%)
GG4	44 (7.2%)
GG5	77 (12.6%)
Stage*	
T1–T2	440 (72.0%)
T3–T4	171 (28.0%)
Cambridge Prognostic Group*	
CPG1	119 (19.4%)
CPG2	152 (24.9%)
CPG3	114 (18.7%)
CPG4–5	206 (33.7%)
Metastatic at diagnosis	20 (3.3%)

**Table 2:** Description of the biomarkers tested and the numbers of data points available for each. PSA, prostate-specific antigen; PSA density, prostate-specific antigen divided by prostate volume

Biomarker variable tested	
PSA density (ng/mL^2^) (*n* = 782)	
Median	0.16
Interquartile range	0.10–0.26
Free to total PSA ratio (*n* = 678)	
Median	0.11
Interquartile range	0.08–0.15
Prostate Health Index (*phi*) score (*n* = 284)	
Median	41.9
Interquartile range	29.1–64.0
Polygenic risk score (PRS) (*n* = 798)	
Median	20.6
Interquartile range	20.0–21.1
MRI Likert/PIRAD score (*n* = 798)	
No lesion	229 (28.7%)
3	130 (16.3%)
4–5	437 (54.8%)
MRI contraindicated	2 (0.02%)

### Performance in prediction of disease detection

#### Polygenic risk scores (PRS)

PRS in this study did add predictive value to detection of any cancers when compared to the base model of PSA + Age in both the pre-MRI setting (AUC 0.73 vs 0.65, *p* < 0.00001) (Additional File: Table S1A) and post-MRI setting (AUC 0.79 vs 0.74, *p* = 0.0002) (Additional File: Table S1B). However, PRS scores did not add incremental value in predicting ≥ GG2 or ≥ GG3 disease in the pre-MRI setting (AUC 0.68 vs 0.66 and 0.69 vs 0.68, *p* = 0.08 and *p* = 0.59 respectively) (Table 3). Using detection of ≥ CPG2 disease as the endpoint in the pre-MRI setting showed some modest benefit (AUC 0.73 vs. 0.70) but not for detection of ≥ CPG3 disease (0.76 vs. 0.75, *p* = 0.16). In the post-MRI setting (MRI Likert ≥ 3), this lack of benefit for significant cancer detection was also evident with only marginal and mostly non-significant gains in AUC (Table 4). These data do not appear to support use of PRS in reliably predicting significant cancer presence in a PSA-elevated population. The PRS panel used in particular did not add value in detection of ≥ GG3 or ≥ CPG3, i.e. higher grade and poorer prognosis disease.

**Table 3:** Additional value of individual test to improve AUC base performance for detection of cancer using different definitions of significance BEFORE an MRI is added. Base model is standard of care including PSA, age. PRS, polygenic risk score; FTPSA, free to total PSA; PSAd, PSA density; *phi*, Prostate Health Index

	≥ GG2 cancer detection	*p* value vs base model	≥ GG3 cancer detection	*p* value vs base model	≥ CPG2 cancer detection	*p* value vs base model	≥ CPG3 cancer detection	*p* value vs base model
Pre-MRI base model (PSA + age)	0.66 (0.62–0.70)	-	0.68 (0.64–0.72)	-	0.70 (0.66–0.74)	-	0.75 (0.72–0.78)	-
Pre-MRI base model + PRS	0.68 (0.65–0.72)	0.08	0.69 (0.64–0.73)	0.59	0.73 (0.70–0.77)	0.0068	0.76 (0.73–0.79)	0.16
Pre-MRI base model + FTPSA	0.71 (0.67–0.74)	0.016	0.69 (0.65–0.73)	0.28	0.75 (0.71–0.78)	0.002	0.76 (0.73–0.80)	0.04
Pre-MRI base model + PSAd	0.74 (0.73–0.80)	< 0.00001	0.73 (0.69–0.77)	0.0006	0.77 (0.74–0.80)	< 0.00001	0.79 (0.76–0.83)	< 0.00001
Pre-MRI base model + *phi*	0.82 (0.76–0.87)	< 0.00001	0.82 (0.76–0.88)	0.0002	0.83 (0.78–0.87)	< 0.00001	0.82 (0.77–0.87)	< 0.00001
Pre-MRI base model + FTPSA + PSAd	0.75 (0.72–0.79)	< 0.00001	0.73 (0.69–0.77)	0.0004	0.78 (0.75–0.82)	< 0.00001	0.80 (0.77–0.83)	< 0.00001

**Table 4:** Additional value of individual test to improve AUC base performance for detection of cancer using different definitions of significance AFTER an MRI is added. Base model is standard of care including PSA, age, MRI LIKERT ≥ 3. PRS, polygenic risk score; FTPSA, free to total PSA; PSAd, PSA density; *phi*, Prostate Health Index

	≥ GG2 cancer detection	*p* value vs base model	≥ GG3 cancer detection	*p* value vs base model	≥ CPG2 cancer detection	*p* value vs base model	≥ CPG3 cancer detection	*p* value vs base model
Base model (PSA + age + MRI LIKERT 3–5)	0.72 (0.68–0.75)	-	0.71 (0.67–0.75)	-	0.75 (0.72–0.79)	-	0.78 (0.74–0.81)	-
Base model + PRS	0.73 (0.69–0.76)	0.21	0.71 (0.67–0.75)	0.48	0.77 (0.74–0.81)	0.017	0.79 (0.75–0.82)	0.16
Base model + F/T PSA	0.75 (0.71–0.78)	0.004	0.71 (0.67–0.75)	0.33	0.79 (0.76–0.82)	0.0002	0.79 (0.76–0.82)	0.013
Base model + PSAd	0.76 (0.73–0.80)	< 0.00001	0.74 (0.70–0.78)	0.0026	0.80 (0.76–0.83)	< 0.00001	0.81 (0.78–0.84)	0.0001
Base model + *phi*	0.84 (0.79–0.88)	0.0001	0.84 (0.78–0.89)	0.0025	0.85 (0.81–0.89)	0.0001	0.86 (0.81–0.90)	0.0015
Base model + F/T PSA + PSAd	0.78 (0.74–0.81)	< 0.00001	0.74 (0.70–0.78)	0.0018	0.81 (0.78–0.84)	< 0.00001	0.82 (0.78–0.84)	< 0.00001

#### Free to total PSA ratio (FTPSA)

FTPSA, in common with all the biomarkers tested in this study, added some value in predicting detection of any cancer (Additional File: Table S1A and B), although incremental benefit did not match those of PSAd or *phi*. For detection of ≥ GG2, FTPSA only added a modest incremental benefit in the pre-MRI context (AUC 0.71 vs.0.66) but no significant enhanced value for ≥ GG3 detection (AUC 0.69 vs. 0.68, *p* = 0.59) (Table 3). In detection of ≥ CPG2, adding FTPSA provided an increase in AUC (0.75 vs. 0.70) but again only a small incremental value in detection of ≥ CPG3 disease (AUC 0.76 vs. 0.75). In the post-MRI setting (MRI Likert ≥ 3), FTPSA did show some incremental value in detection of ≥ GG2 (AUC 0.75 vs. 0.72) and ≥ CPG2 (0.79 vs. 0.75) disease but not for ≥ GG3 or ≥ CPG3 disease (Table 4). Thus, overall, like for PRS, the addition of FTPSA failed to show much benefit over PSA + Age or PSA + Age + MRI Likert ≥ 3 base especially in detection of higher-grade/poorer prognosis disease.

#### PSA density (psad)

PSAd demonstrated consistent improvement in detection of any cancer and significant cancers regardless of definition used in the pre-MRI context (all comparisons *p* < 0.001) (Additional File: Table S1A and Table 3). Notably, the incremental gain was highest when the end point was detection of composite prognostic detection endpoints, i.e. ≥ CPG2 (AUC 0.77 vs. 0.70) and ≥ CPG3 disease (0.79 vs. 0.75) (Table 3). Of note, PSAd added to the base model of PSA + Age performed similarly to a base model of PSA + Age + MRI Likert ≥ 3 in detection of ≥ GG2, ≥ GG3, ≥ CPG2 and ≥ CPG3 disease (Tables 3 and 4). The added value of PSAd was also evident in the post-MRI setting regardless of endpoint definition (Table 4). Here again, the incremental value was highest when the end point was a composite prognostic endpoint: ≥ CPG2 (AUC 0.80 vs. 0.75) and ≥ CPG3 disease (0.81 vs. 0.78) (Table 4). Further adding FTPSA to PSAd did not add incremental value in either the pre- or post-MRI models (Tables 3 and 4).

#### Prostate health index (phi)

Of all the biomarkers test, *phi* added the greatest incremental value for any cancer detection scenario with AUC consistently high for any cancer or significant cancer endpoints in both the pre-MRI and post-MRI setting (Additional File: Tables S1 A and B, Tables 3 and 4). In the pre-MRI context, performance was equally strong for detection of ≥ GG2 and ≥ GG3 (AUC 0.82 and 0.82 vs. 0.66) disease as it was for detection of ≥ CPG2 and ≥ CPG3 disease (AUC 0.83 and 0.82 vs. 0.66) (*p* < 0.0001 for all comparisons). Importantly, including *phi* in the pre-MRI base model outperformed the predictive accuracy afforded by using MRI Likert ≥ 3 for detection of ≥ GG2, ≥ GG3, ≥ CPG2 and ≥ CPG3 disease (Tables 3 and 4). Adding *phi* to the post-MRI model (PSA + Age + MRI Likert ≥ 3) further improved overall performance achieving AUC of 0.84–0.86.

### Decision curve analysis (DCA) in reducing over-investigation

**Fig. 1:**
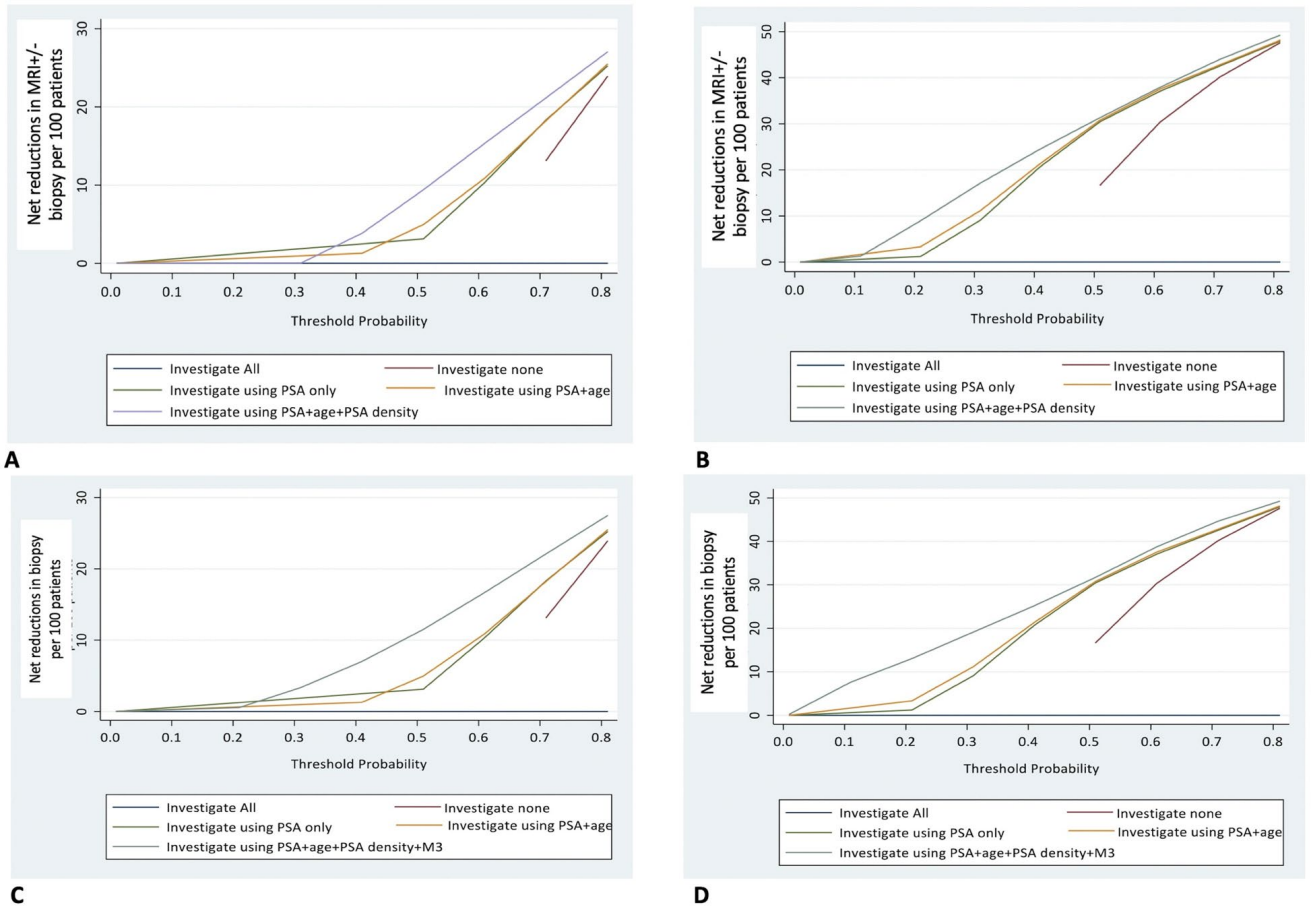
Decision curve analysis comparing the net reduction in investigations needed when including PSA density (PSAd) and then MRI (M3–PIRADS ≥ 3) for a range of risk threshold values with **A** ≥ Cambridge Prognostic Group 2 (CPG2) as a detection endpoint and reducing both MRI ± biopsy events, **B** ≥ Cambridge Prognostic Group 3 (CPG3) as a detection endpoint and reducing both MRI ± biopsy events, **C** ≥ CPG2 as a detection endpoint and reducing biopsy events and **D** ≥ CPG3 as a detection endpoint and reducing biopsy events

**Fig. 2:**
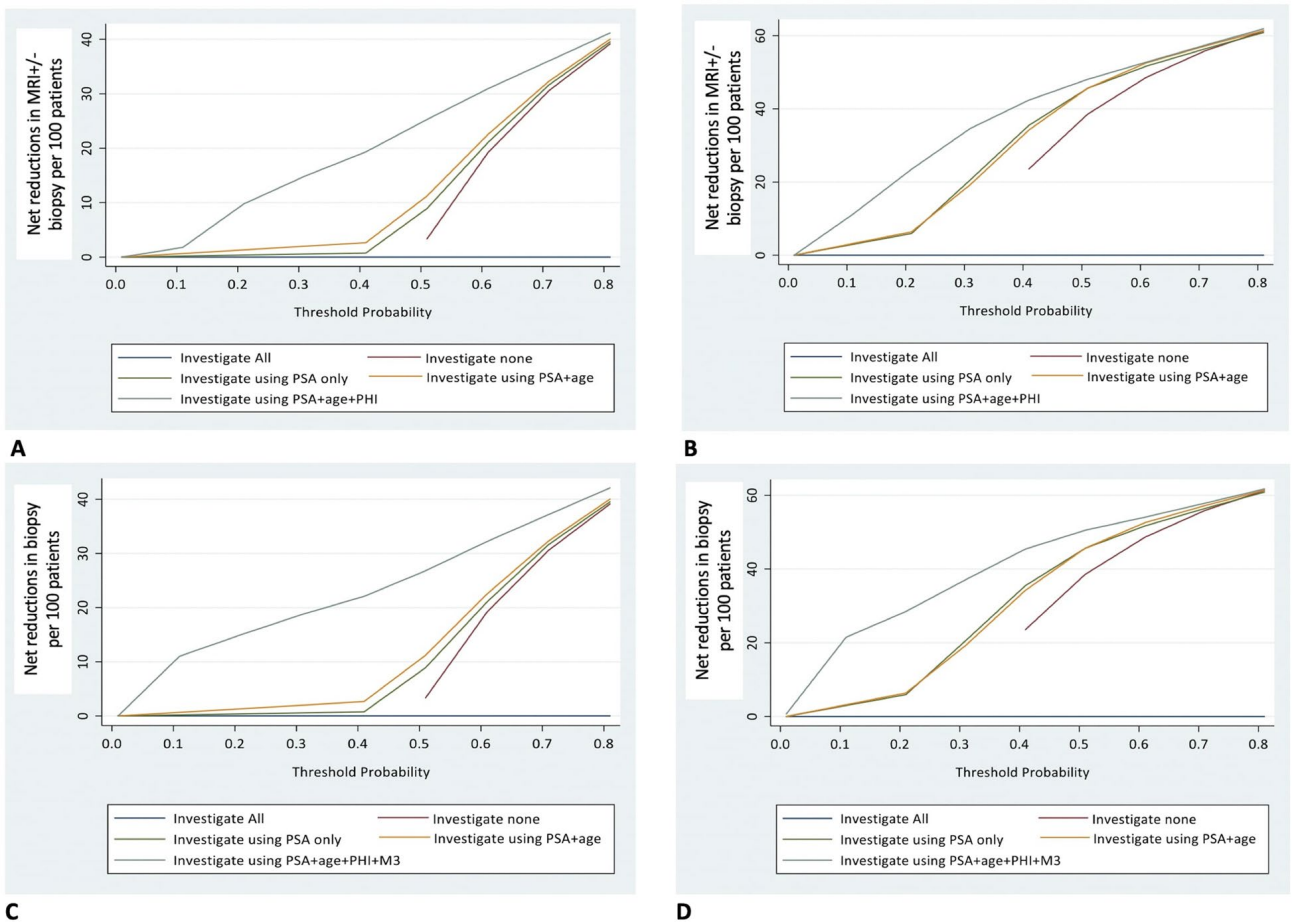
Decision curve analysis comparing the net reduction in investigations needed when including Prostate Health Index (*phi*) and then MRI (MR3–LIKERT ≥ 3) for a range of risk threshold values with **A** Cambridge Prognostic Group 2 (CPG2) as a detection endpoint and reducing both MRI ± biopsy events, **B** Cambridge Prognostic Group 3 (CPG3) as a detection endpoint and reducing both MRI ± biopsy events, **C** CPG2 as a detection endpoint and reducing biopsy events and **D** CPG3 as a detection endpoint and reducing biopsy events

To understand the value in reducing over-investigation, we generated DCA for PSAd and *phi*, the 2 most promising biomarkers in our study. Using PSAd and with ≥ CPG2 as an endpoint, above a threshold of 30% ( without missing cancer) using PSA + Age + PSAd dominated with net reductions in MRI and the need for subsequent biopsies which is probably too high a risk in routine clinical practice (Fig. 1A). With ≥ CPG3 as an endpoint, the net benefit however was gained at much lower threshold probabilities (≥ 10%) (Fig. 1B). In models to only reduce biopsies (post-MRI), the dominant model was a combination of PSA + Age + PSAd + MRI Likert ≥ 3, with net reductions in biopsies above threshold probabilities of 20% (Fig. 1C). With ≥ CPG3 as an endpoint, the net benefit of PSA + Age + PSAd + MRI ≥ 3 was evident regardless of the threshold probability and dominated all other models (Fig. 1D). With the *phi* assay, it was clear that all models which incorporated *phi* were effective at reducing use of both MRI and biopsies. In the pre-MRI setting, whether the endpoint was detection of ≥ CPG2 or ≥ CPG3, use of *phi* resulted in a net reduction in number of investigations across all risk thresholds (Fig. 2A, B). Similarly, in models to only reduce biopsies, the dominant model was a combination of PSA + Age + *phi* + MRI Likert ≥ 3, with net reductions in biopsies evident across all threshold probabilities and in detection of both ≥ CPG2 or ≥ CPG3 disease (Fig. 2C, D). Taken together, these results suggest that PSAd and *phi* may be useful to refine and reduce use of MRI and biopsies as part of a stepwise tiered diagnostic pathway, but this is dependent on the definition of significance used.

## Discussion

In this study, we compared and modelled different additional biomarkers to refine the predictive value of the ubiquitous PSA test. We find that the performance of biomarkers added to PSA differs based on the definition of clinical significance used and also whether in the pre- or post-MRI context. We further identified that using a composite definition of significant cancer based on prognosis resulted in better biomarker performance and a more contextualised value for decisions to investigate. Consistently, all biomarkers were able to better detect composite CPG2 and CPG3 disease compared to using a histological grade group endpoint alone. MRI has been a game changer in modern prostate diagnostics but remains a resource expensive and interpreter-dependent tool. Maintaining high-quality services requires regular quality assessment and standardisation [13]. In contrast, liquid biomarker tests are less subjective and simpler to adopt and standardise, with the caveat that  measurements may vary between providers using different biochemical assay brands [14]. Considering the health resource needs of the future as well as environmental imperative to reduce energy consumption, it is important to identify tests that can refine and reduce the use of imaging as well as biopsy [15]. In our opinion, the MRI and biopsy should therefore be considered a single integrated tertiary referral endpoint of the diagnostic pathway.

To our knowledge, this is one of the first studies to explore the relative comparative value of different tests including genomics, serum-based markers and imaging within the same cohort. Our work suggests that a primary additional value is gained by serum biomarkers based on PSA isoforms and prostate volume. The *phi* and PSA density tests consistently improved the predictive ability to detect significant cancer regardless of the endpoint definition. Indeed, use of these biomarkers added to base models of PSA + Age outperformed MRI. However, it is worth noting that the DCA showed that the benefit gain for PSAd depends very much on the definition of clinical significance used and context (i.e. whether trying to reduce MRI and biopsies or biopsies alone). As an example, a biopsy threshold of ≥ 30% is too high for normal standard practice for missing CPG2 cancers (if this was the definition of clinical significance used). However, when using CPG3 as a definition of clinical significance, the threshold is 10%, which is likely more acceptable for clinical practice. For *phi*, the thresholds were all lower (10% or less) across different scenarios (reducing MRI and/or biopsies and whether pre- or post-MRI) and for different definitions of clinical significance. Free to total PSA performance was variable and added only modest incremental value especially in detection more aggressive cancers. Our data also suggest that PRS likely do not add much value in improving the detection of significant cancers, at least, in men already referred with a raised PSA. Most previous studies have either looked at individual variables or a fixed panel. Perhaps the most well-known combination of the latter is the SThM3 test which includes PSA, different kallikreins, genomic SNPs and molecular markers as well as clinical factors [16]. These are combined into an algorithmic score to predict the likelihood of cancer (defined as histological grade ≥ GG2). In a non-MRI population, the AUC achieved was 0.74 versus 0.5 with PSA alone, not dissimilar to some of the individual biomarkers in this present study [16]. Subsequent works in MRI-imaged populations have shown similar incremental value gains in AUC [17]. Strom et al. published the individual contributions of each component to the overall performance of the SThM3 test [18]. Clinical variables (i.e. prostate volume, previous biopsies, and prostate volume) added the most to the cumulative AUC with prostate volume producing the greatest increase. Notably, individual molecular markers and genomic scores added very little to the test’s final predictive ability, and this is consistent with our own findings here (18).

The definition of significant cancer and its impact on biomarker performance has not been previously well explored in early detection research. The standard endpoint is usually based on histological grade (≥ GG2). Yet, the clinical validity of this endpoint is debatable especially as there is good evidence that many GG2 cancers (especially if detected by MRI targeting) also can have a very slow natural history [19, 20]. Moreover, prognosis (the risk of dying of cancer) is long established to be based on not just histopathology alone but also on stage and PSA level and is what is used in clinical decision making after a diagnosis [6, 21]. Indeed, in nearly all prostate cancer guidelines, management is recommended based on risk/prognostic group rather than just grade group [11, 22]. Hence, it is logical to consider a composite endpoint based on prognosis as the definition of significance as this directly informs clinical decision making and improved survival outcomes (i.e. the raison d’etre for any early detection or screening programmes). In this study, when CPG2 and CPG3 were used as an endpoint, it was notable that the performance of liquid biomarkers as well as imaging all improved. Previous work from our unit has demonstrated that ≥ CPG3 disease really represents when prostate cancer treatment has a significant impact in reducing mortality [23]. We therefore advocate that a detection endpoint of ≥ CPG3 disease is the most useful for prostate cancer detection strategies. Our modelling would further suggest that using this endpoint was particularly useful in differentiating who needs to proceed to an MRI and, if needed, to subsequent biopsies.

Our study does have limitations being a retrospective series based on an already referred population with elevated PSA. This was thus a selected population and relatively small cohort with little racial diversity. It may also not be applicable to cohorts with lower cancer prevalence. It does therefore need replication in larger studies and further testing in the context of early detection/screening. In this regard, there are currently initiatives looking at new prostate cancer detection methods, and we trust our data will be of use in informing their efforts [24]. Our cohort of men had biopsies regardless of MRI lesion visibility, so we know the true  cancer prevalence without excluding men with a negative MRI. Despite this, we cannot rule out occult selection bias we were not aware of as men were seen by different clinical team members. We also focused on PRS and not pathogenic genetic variants, which could potentially improve the predictive accuracy of genomics. This work is also based on the current available PRS, and there are ongoing initiatives to find improved PRS. *phi* data was also only available in a sub-cohort of men, but our findings do mirror that of many other papers who have reported on its strong predictive performance and ability to refine referrals [25]. We further note that other biomarkers have also recently shown some value in pre-MRI prostate triage testing in the screening setting, e.g. the recently reported Pro-Screen study incorporating the 4K panel [26]. Our work also does support and underpin efforts in the European Union (amongst others) to incorporate PSAd into organised prostate testing programmes [27]. The key question is how prostate volume (to derive the PSA density) could be reliably measured in a community clinic setting and before an MRI. Other research from our unit has now also addressed this with a recent study that has shown that prostate volumes (comparable to MRI) can be reliably measured using standard non-invasive surface measurements with transabdominal/transperineal ultrasound (US) [28]. Sensitivity of 100% and specificity 85% were obtained in our study, and US could even be done using very low-cost hand-held devices now available.

## Conclusions

In summary, we present data comparing different biomarkers strategies to increase discrimination of cancer detection in a PSA-referred population. We find no place for PRS but identify that PSAd and *phi* especially can reduce and refined the use of MRI and biopsies. PSAd offers a low to no cost option for a reflex test to refine onward referral for imaging/biopsy. Importantly, the incremental gain using these markers is strongly dependent on the definition of significant cancers used as the endpoint for detection. In this regard, we propose the use of a composite prognostic endpoint classification (e.g. ≥ Cambridge Prognostic Group 3) that mirrors the post-diagnosis classification used for decision making. Taking all these together, we advocate that the future of prostate cancer detection should adopt a tiered strategy with each test forming a gateway to the next investigation. In this scenario, the burden of over-imaging and over-biopsy can be simultaneously addressed while enriching detection of prognostically important disease.

## Supplementary Information

Additional File. Table S1. Additional value of individual test to improve AUC Base performance for detection of any cancer. A. Base model is pre-MRI standard of care including PSA, age. B. Base model with post-MRI standard of care including PSA, age, MRI and biopsy of Likert 3-5. All p value are comparisons against the base models. PRS- polygenic risk score FTPSA- Free to total PSA PSAd- PSA density, phi -Prostate Health Index.

## Data Availability

All data is available by application to the study authors.

## Abbreviations

PSA: Prostate-specific antigen
FTPSA: Free/total PSA
*phi*: Prostate Health Index
PSAd: PSA density
PRS: Polygenic risk score
GG: Grade group
CPG: Cambridge Prognostic Group
AUC: Area under the curve
DCA: Decision curve analysis
MRI: Magnetic resonance imaging
SNP: Single-nucleotide polymorphism
PI-RADS: Prostate Imaging Reporting and Data

## Data availability

Data is available on request and applications to the authors based on the authors institutional requirements.
