# Supplementary material for: The efficacy of different biomarkers and endpoints to refine referrals for suspected prostate cancer: the TARGET study (Tiered integrAted tests for eaRly diaGnosis of clinically significant ProstatE Tumours)
Source: BMC Med. 2024 Oct 8;22:440. doi: 10.1186/s12916-024-03667-7 (PMC11462681; doi:10.1186/s12916-024-03667-7)
Supplement: Supplementary file 1 — Additional File. Table S1. Additional value of individual test to improve AUC Base performance for detection of any cancer. A. Base model is pre-MRI standard of care including PSA, age. B. Base model with post-MRI standard of care including PSA, age, MRI and biopsy of Likert 3-5. All p value are comparisons against the base models. PRS- polygenic risk score FTPSA- Free to total PSA PSAd- PSA density, phi -Prostate Health Index. [file 12916_2024_3667_MOESM1_ESM.docx]

|  | **Any cancer** | **p value**  **vs base model** |
| --- | --- | --- |
| **Pre-MRI Base Model**  **(PSA + Age)** | 0.65  (0.60-0.69) | - |
| **Pre-MRI Base model +PRS** | 0.73  (0.69-0.77) | <0.00001 |
| **Pre-MRI Base model +FTPSA** | 0.68  (0.64-0.72) | 0.018 |
| **Pre-MRI Base model + PSAd** | 0.75  (0.71-0.79) | <0.00001 |
| **Pre-MRI Base model + *phi*** | 0.83  (0.77-0.88) | <0.00001 |
| **Pre-MRI Base model +F/T PSA+PSAd** | 0.76  (0.72-0.79) | <0.00001 |

**A**

|  | **Any cancer** | **p value**  **vs base model** |
| --- | --- | --- |
| **Base Model**  **(PSA + Age + MRI LIKERT 3-5)** | 0.74  (0.70-0.78) | - |
| **Base model +PRS** | 0.79  (0.75-0.83) | 0.0002 |
| **Base model +FTPSA** | 0.76  (0.72-0.79) | 0.08 |
| **Base model + PSAd** | 0.79  (0.75-0.82) | <0.00001 |
| **Base model + *phi*** | 0.86  (0.81-0.90) | 0.0006 |
| **Base model +F/T PSA+PSAd** | 0.79  (0.76-0.83) | <0.00001 |

**B**

**Table S1** - Additional value of individual test to improve AUC Base performance for detection of any cancer. **A.** Base model is pre-MRI standard of care including PSA, age. **B.** Base model with post-MRI standard of care including PSA, age, MRI and biopsy of LIKERT 3-5**.** All p value are comparisons against the base models. PRS- Polygenic risk score FTPSA- Free to total PSA PSAd- PSA density, *phi* -Prostate Health Index
